# Infected food web and ecological stability

**DOI:** 10.1038/s41598-022-11968-1

**Published:** 2022-05-17

**Authors:** Akihiko Mougi

**Affiliations:** Institute of Agricultural and Life Sciences, Academic Assembly, Shimane University, 1060 Nishikawatsu-cho, Matsue, 690-8504 Japan

**Keywords:** Ecology, Ecology

## Abstract

Parasites are widespread in nature. Nevertheless, they have only recently been incorporated into food web studies and community ecology. Earlier studies revealed the large effects of parasites on food web network structures, suggesting that parasites affect food web dynamics and their stability. However, our understanding of the role of parasites in food web dynamics is limited to a few theoretical studies, which only assume parasite-induced mortality or virulence as a typical characteristic of parasites, without any large difference in terms of predation effects. Here, I present a food web model with parasites in which parasites change the mortality and interaction strengths of hosts by affecting host activity. The infected food web shows that virulence and infection rate have virtually no effect on food web stability without any difference in interaction strengths between susceptible and infected individuals. However, if predation rates are weakened through a restriction of the activity of infected individuals, virulence and infection rate can greatly influence stability: diseases with lower virulence and higher transmission rate tend to increase stability. The stabilization is stronger in cascade than random food webs. The present results suggest that parasites can greatly influence food web stability if parasite-induced diseases prevent host foraging activity. Parasite-induced infectious disease, by weaking species interactions, may play a key role in maintaining food webs.

## Introduction

Parasites are widespread in ecosystems, and can account for a large part of the biomass on earth^1,2^. Nevertheless, parasites have only recently begun to be considered in community ecology^3–13^. Earlier studies showed that the effects of including parasites in food webs are so large that they modify network topology by increasing the connectance and food-chain length^14,15^. Because of the strong relationship between food web topology and population dynamics^16,17^, parasites may affect community stability^14,15^. On the other hand, as shown in classical epidemiological theories, parasites can regulate host populations^18,19^ and, therefore, can affect community and ecosystem dynamics^8,20^. In spite of the potential large effects of parasites on community dynamics, few theoretical studies have examined how parasites affect the stability of such complex communities, particularly with diverse species and their interactions^21,22^.

Most earlier efforts to understand the effects of parasites on community dynamics used simple systems with a few species, such as two host-one parasite models^23,24^. Hence, we know very little about the roles of parasites within large ecological communities with diverse species. Still, a pioneering work, which considered the dynamics of multiple hosts infected by a parasite, demonstrated a role of host diversity in the establishment, persistence, and outbreak of the parasite, but without considering any direct interaction between host species^25^. A few theoretical studies have introduced parasites into a food web with direct interactions between species^21,22^. Contrary to the intuitive view that parasites can contribute to community stability through host population regulation, parasites almost never affect food web stability^21,22^. Rather, parasites may even contribute to food web instability because parasite invasion can sometimes induce population cycles^21^. In earlier infected food web models, a key role of parasites was to regulate host population growth by increasing host mortality (virulence)^21,22^. However, this host regulation effect is a similar feature of predators in food webs; thus, the unique features of the parasite itself must be considered to deeply understand the roles of parasites in food web dynamics^6,15^.

Parasites can affect other important characteristics of hosts, such as species interactions and reproduction^15,24^. The most intuitive example is inactivity due to an infectious disease, through which infected individuals become less able to capture and/or eat prey resources, and breed and/or lose reproductive ability^24^. In addition, ailing individuals are expected to be more easily captured by predators^24^. Thus, parasites, through the infectious disease itself, can change the network structure of food webs in terms of affecting interaction strengths. If so, the parasite-induced infectious disease could largely affect the food web dynamics, because interaction strength plays a key role in food web stability^26,27^.

**Figure 1:**
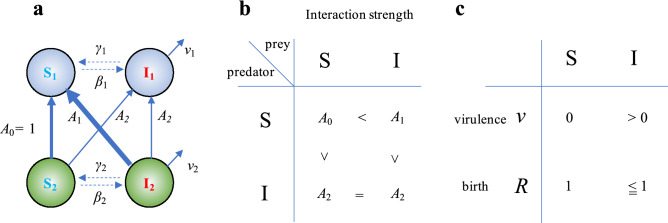
Schematic diagram of the simplest infected food web. (**a**) Model structure. S_i_ and I_i_ represent a susceptible and infected individual, respectively (*i* = 1; predator, *i* = 2; prey). *A*_0_ is the normalized interaction strength among susceptible predators and prey. *A*_1_ and *A*_2_ are the relative strength of interaction among predators and prey, indicated by each arrow to *A*_0_. Each species has a disease with transmission rate *β*_*i*_, virulence *v*_*i*_ and recovery rate *γ*_*i*_. (**b**) Effects of the disease to interaction strengths between predator and prey. The strength of interactions are represented by the arrow thickness in (**a**). (**c**) Effects of disease to birth and death rates. In the main text, birth rate is same among susceptible and infected individuals, although the effect of birth reduction due to disease is shown in Supplemental Information.

In this paper, using a food web model with a disease, I show that disease-induced changes in the strengths of species interactions play a key role in stabilizing complex communities. I consider a cascade food web model^28^ comprising *N* species, any pair of which is connected with a probability of *C* (connectance). For each pair of species, *i*, *j* = 1,…, *N* with *i* < *j*, species *i* never consumes species *j*, whereas species *j* may consume species *i.* The infectious disease is caused by a specialist parasite to each host species (coinfection and cross-species infection are not considered). Once a disease spreads, each population has susceptible or uninfected (S) and infected (I) individuals. The infection occurs through contact with infected individuals, where the infection rate of each species is notated by *β*_*i*_, and infected individuals recover at a constant rate *γ*_*i*_ into susceptible individuals. The parasites do not increase through vertical transmission. The infected individuals have a higher death rate than susceptible individuals; the additional death rate is defined as virulence, notated by *v*_*i*_. Population dynamics are driven by interspecific predator–prey interactions and host-parasite interactions via the infectious disease (see Methods). The interaction strength is a key factor affecting the food web dynamics. Here, I consider that the interaction strengths are changed by the disease; to do this, I control the relative interaction strength to a basal predator–prey interaction in a food web model without diseases. Each interaction strength is normalized by the interaction strength among S (predator) vs. S (prey). The relative interaction strength among S (predator) vs. I (prey) is defined as *A*_1_, and that of I is defined as *A*_2_ (Fig. 1a). A typical expectation of the changes in interaction strength due to diseases would be weak interactions of infected individuals and/or strong interactions of susceptible individuals to infected individuals (Fig. 1b). In addition to an increase in mortality due to a disease, reproduction rate can be decreased. The reduction of the reproduction is controlled by a parameter, R, which is a constant that controls the degree of growth rate reduction from the susceptible to infected individuals. In this study, I mainly control the relative interaction strengths (*A*_1_ and *A*_2_) to examine how diseases affect the stability of food webs, as evaluated via community persistence, with the probability that all species persist for a given time (see Methods for details).

## Results

Consider a healthy food web without diseases (*β*_*i*_ = 0). In such a situation, it is difficult for complex communities with diverse species to persist, as well known from previous food web models^29,30^. This suggests that a healthy world is unstable. However, in nature, there are no healthy ecosystems without parasite-induced infectious diseases.

**Figure 2:**
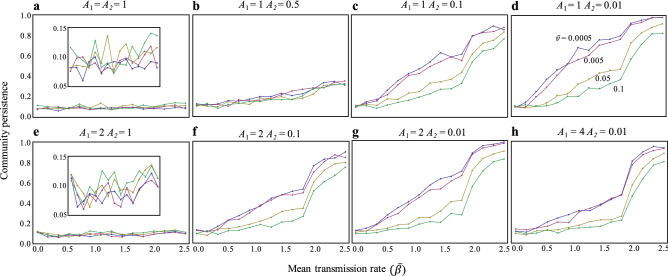
Effects of virulence and infection rate on community stability with varying interaction strengths. Each figure (**a**–**h**) has different interaction strength values, as indicated in the upper side of each panel. Color represents the mean virulence values. N = 30 and C = 0.3. Parameters are *R* = 1, $\overline{r}$ = 1, $\overline{d}$ = 0.005, $\overline{a}$ = 0.05, and $\overline{\gamma }$ = 0.005.

Here, consider the introduction of parasites or pathogens into the food web (*β*_*i*_ > 0), which induces infectious diseases in each species. This infection can increase host mortality (virulence effect). When the infectious disease only has the virulence effect, the virulence level has not a significant effect to stability (Fig. 2a), although higher virulence can slightly increase stability when infection rate is high (Fig. S1); this suggests that virulence due to an infectious disease does not play a significant role in food web stability.

**Figure 3:**
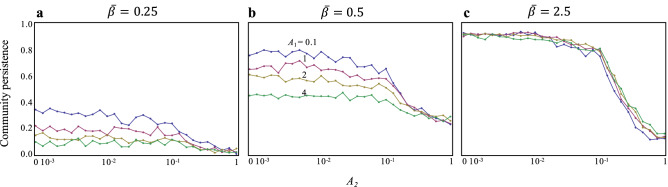
Effects of interaction strengths on community stability with varying infection rates. (**a**) $\overline{\beta }$ = 0.25. (**b**) $\overline{\beta }$ = 0.5. (**c**) $\overline{\beta }$ = 2.5. Color represents values of *A*_1_. Other information is identical to that in Fig. 2.

However, this is not true if predator–prey interactions are altered by infection; this can change predation rates. First, when the predation rates of infected individuals are weaker than those of susceptible individuals (*A*_1_ > *A*_2_), infection rate can largely affect community stability, with stability increasing as infection rates increase (Fig. 2b). In addition, as the predation rates of infected individuals become weaker (*A*_1_ > > *A*_2_), virulence beings to affect stability, wherein a higher virulence tends to decrease stability (Fig. 2c,d). These patterns are qualitatively held, even when the predation rates of susceptible individuals to infected individuals are stronger than those of susceptible individuals (*A*_1_ > 1; Fig. 2e–h). On the other hand, the effects of the predation rates of susceptible individuals to infected individuals (*A*_1_) on stability depend on the infection rate. When infection rates are low, higher values of *A*_1_ tend to decrease stability (Fig. 3a,b); however, when infection rates are high, the values of *A*_1_ almost never affect stability (Fig. 3c). These results are not affected by the reproduction rates of infected individuals. Infertility due to infection does not change the results, although infertility can have a somewhat stabilizing effect when the infection rate is high and the disease has a lower effect on interaction strengths (Fig. S2). The recovery rate tends to decrease stability (Fig. S3), suggesting that the continuous emergence of infected individuals with a loss of appetite plays a key role in food web stability. The stability of infected food webs is so strong that it is almost never reduced by an increase in food web complexity, species richness, and connectance, contrary to a food web without infection (Fig. S4).

I also tested other food web models: an interspecific infection model, a random network model, a free-living parasite model and vertical transmission model (Supplemental Online text). Interspecific infection causes stability in two ways, depending on the intraspecific infection rate. The stability tends to increase with an increase in interspecific infection rate when intraspecific infection rate is low, whereas the tendency is reversed when intraspecific infection rate is high (Fig. S5). Contrary to the cascade model, random food webs show lower stability (Fig. S6). In addition, with a large *A*_1_, an increase in infection rate can decrease stability, particularly when *A*_2_ or virulence is large. This instability is so large that high values of *A*_1_ decrease stability even when infection rates are high (Fig. S6). On the other hand, a difference in the assumption on an infection process does not change the qualitative results. I consider a model in which infection occurs only through contact with a free-living parasite (Supplemental Online text); even in this case, the main results are qualitatively held (Fig. S7). Further, vertical transmission can contribute to stability, although the contribution is not significant (Fig. S8).

## Discussion

The present study showed that a parasite-induced infectious disease can play a key role in stabilizing food web dynamics via a change in interaction strengths caused by the disease. If parasites do not cause any difference in traits, other than mortality to the infected host individuals, parasites almost do not affect food web stability. However, once the parasite-induced infectious disease can influence the interaction strengths, the difference in interaction strengths among susceptible and infected individuals allow the key characteristics of infectious diseases (infection rate and virulence) to have an effect on food web stability, wherein a higher infection rate and lower virulence can contribute to food web stability. The stabilizing effect of an infectious disease becomes large when infection causes a loss of appetite or decreases predation rates. This stabilizing effect does not depend on whether parasites cause infertility nor have a free-living period, but it does depend on the food web network structure. Infectious disease is likely to have a more stabilizing role in a cascade food web than in a random food web. These results suggest that the suppression of activity caused by infectious diseases can play a key role in stabilizing natural food webs.

Why can infected food webs be stable? Infectious diseases inherently have two stabilizing effects. First, less active, infected predators can prevent a large reduction in the number of prey. If infection rates are high, abundant predators can be suppressed by the infectious disease due to disease-induced mortality and appetite loss^24,31^. The predator suppression due to disease can prevent a large reduction in the number of prey. In addition, predation of infected prey can also suppress the infectious disease in the prey, resulting in the prevention of a reduction of the prey. Because the number of prey is not reduced as normal, infected prey can also act as an alternative resource for susceptible predators^32^, preventing a large reduction in the number of predators. However, susceptible predator is not likely to be more abundant as explained above, the predation effect is maintained at a low level (because the consumption rare itself does not change but predator abundance is low). As a consequence, both predator and prey are unlikely to be less and more abundant, resulting in a stabilization of population dynamics. This suggests a disease induced self-maintenance mechanism in food webs. However, the mechanism is not likely to stabilize when the food web structure is not a cascade (i.e., random). In a cascade network, top predators and basal prey resources always exist. Since a top predator is never eaten by another species, an abundant predator is likely to be regulated by the disease. On the other hand, in a random network, top predators and/or basal prey species may not exist; if so, susceptible predators become less abundant because of predation from other species. Altogether, this results in the prevention of predator regulation and leads to destabilization of the food web.

Parasites or infectious disease-induced interaction change are critical in leading a stabilizing role in food web dynamics; this has been supported by a simple predator–prey system with a parasite^31,33^, but it has never been demonstrated in more complex systems with diverse species. The present study suggests that revealing a network structure of infected food webs with respect to interaction strengths is important for understanding the role of infectious diseases in ecological communities. Conversely, from a community stability standpoint, we need to focus on the parasites that alter interaction structures, among all diverse parasites. On the other hand, the present model is not applicable to all types of parasites; however, it is at least applicable to a disease occurring through intra- and interspecific infection or contact with a free-living parasite. Interspecific infection can promote the stabilization of an infectious disease when intraspecific infection is less frequent since it can merely contribute to increasing the number of infected individuals. However, when both intra- and interspecific infection rates are high, the extinction of less abundant susceptible individuals would prevent a high stabilization effect. Nevertheless, examining the types of parasites that largely affect ecological community stability will be an important future research area.

May mathematically demonstrated that complex communities comprised of diverse species with many interaction links are inherently unstable^29,30^. As this prediction was in contrast to persisting, real ecological communities, ecologists have searched for the key unknown factors of natural ecosystems, and May’s model has not worked. Weak interactions have been suggested to be a major stabilizing factor^26,27^. Real communities appear to be characterized by many weak interactions^34–36^. I propose that infected food webs may be one of mechanisms that cause weak interactions, which can play a key role in maintaining complex communities. A healthy world may be unstable, because in nature, there are no healthy ecosystems without parasite-induced infectious diseases.

## Methods

Consider a cascade food web^28^ in which pairs of species *i* and *j* (*i*,* j* = 1,…, *N*) are connected by a trophic interaction with a probability of *C*, which is defined as the proportion of realized interaction links *L* in the possible maximum interaction links *L*_*max*_ of a given network model (*L* = *CL*_*max*_). For each pair of species, *i*, *j* = 1,…, *N* with *i* < *j*, species *i* never consumes species *j*, whereas species *j* may consume species *i.* To examine the generalization of the main result, random food webs^37^ was also be tested (Fig. S6). The maximum link number *L*_max_ is calculated from *N*(*N* − 1)/2 in both the cascade and random models.

Consider that disease transmission occurs only within each population. Then, each species can have susceptible and infected individuals, denoted by S and I, respectively. Infection occurs horizontally through contact with infected individuals within the same species; the vertical transmission of parasites does not occur. Infected individuals have a higher mortality rate than susceptible individuals. Infected individuals have the same species interaction links with susceptible individuals, whereas interaction strengths and/or reproduction rate will be different from those of susceptible individuals. The food web model with diseases is defined by an ordinary differential equation:

1a $$dX_{Si} /dt = (r_{Si} X_{Si} + r_{Ii} X_{Ii} )(1 - \, X_{i} ) + M_{S} X_{Si} + M_{I}^{ + } X_{Ii} {-}d_{i} X_{Si} {-}\beta_{i} X_{Ii} X_{Si} + \, \gamma_{i} X_{Ii} ,$$

1b $$dX_{Ii} /dt = \beta_{i} X_{Ii} X_{Si} {-}(d_{i} + v_{i} )X_{Si} {-}\gamma_{i} X_{Ii} + M_{I}^{ - } X_{Ii} ,$$

where *X*_*ki*_ is the abundance of species *i* and *X*_*i*_ = *X*_S*i*_ + *X*_I*i*_. The subscript *k* represents the states of individuals, where S and I represent a susceptible and infected host, respectively. *r*_*ki*_ is the intrinsic rate of change in a species *i*, *d*_*i*_ is the basal death rate of species *i*, *v*_*i*_ is the additional death rate due to disease (virulence), *β*_*i*_ is the transmission rate of disease, and *γ*_*i*_ is the recovery rate of disease. *M* is the terms on predator–prey interactions. *M*_S_ = Σ_*j*_*M*_SS*ij*_*X*_S*j*_ + Σ_*j*_*M*_SI*ij*_*X*_I*j*_ represents the population change rate through species interactions of susceptible hosts in species *i*, *M*_I_^+^ = Σ_*j*_*M*_IS*ij*_*X*_S*j*_ + Σ_*j*_*M*_II*ij*_*X*_I*j*_ (> 0) represents the population increase rate through prey consumption by infected host species *i*, and *M*_I_^−^ = Σ_*j*_*M*_SI*ji*_*X*_S*j*_ + Σ_*j*_*M*_II*ji*_*X*_I*j*_ (< 0) represents the population decrease rate through consumption of infected host prey by predators. *M*_*lij*_ is the interaction coefficient between species *i* and *j*. The subscript *l* represents a particular interaction structure. “SS” equates to predator–prey interactions among susceptible host species *i* and *j*, “SI” to predator–prey interactions between susceptible host species *i* and infected host species *j*, “IS” to predator–prey interactions between infected host species *i* and susceptible host species *j*, and “II” to predator–prey interactions among infected host species *i* and *j*. Interaction coefficients are defined as *M*_*lij*_ = *e*_*lij*_*a*_*lij*_ and *M*_*lji*_ =  − *a*_*lij*_, where *a*_*lij*_ is the consumption rate and *e*_*lij*_ (< 1) is the conversion efficiency.

Generally, disease can decrease the activity of a host, such that the consumption rate and/or reproduction rate of infected individuals will decrease. On the other hand, infected individuals can be easily consumed by non-infected predators. To reflect this, for simplicity, I assume that (1) *e*_I*ij*_ = *Re*_*ij*_ (*R* ≤ 1), where *e*_SS*ij*_ = *e*_SI*ij*_ = *e*_*ij*_ and *e*_IS*ij*_ = *e*_II*ij*_ = *e*_I*ij*_; (2) *r*_I*i*_ = *Rr*_*i*_ (*R* ≤ 1), where *r*_S*i*_ = *r*_*i*_; and (3) *a*_SI*ij*_ = *A*_1_*a*_*ij*_ (*A*_1_ ≥ 1) and *a*_I*ij*_ = *A*_2_*a*_*ij*_ (*A*_2_ ≤ 1), where *a*_SS*ij*_ = *a*_*ij*_ and *a*_IS*ij*_ = *a*_II*ij*_ = *a*_I*ij*_. *R*, *A*_1_, and *A*_2_ are constant, which control the relative magnitude of each parameter of infected individuals to that of susceptible individuals.

Parameters in each simulation are randomly determined from a uniform distribution with a lower limit of zero. The default mean values of each parameter in the main text were: $\overline{r}$ = 1, $\overline{d}$ = 0.005, $\overline{a}$ = 0.05 and $\overline{\gamma }$ = 0.005 (hereafter, the bar represents the mean parameter values of all species). For simplicity, I assume *e*_*ij*_ = 0.2. I systematically changed the values $\overline{\beta }$, $\overline{v}$, *R*, *A*_1_, and *A*_2_ to examine the effects of disease on food web dynamics stability. When $\overline{\beta }$ = 0, the system approximated the original food web model without disease. Once $\overline{\beta }$ > 0, the food web was infected by disease. Then, if *R* = *A*_1_ = *A*_2_ = 1, we can see the effects of virulence on food web dynamics. If *R* = 1, but *A*_*i*_ is varied, we can see the effects of disease-induced interaction changes on food web dynamics. If *R* < 1, we can see the effects of infertility due to disease on food web dynamics.

In each iterated simulation, the initial species abundance was randomly selected from a uniform distribution (*X*_S*i*_ = 0–1.0, *X*_I*i*_ = 0–0.01). Community persistence^38^, used as a representative of stability, was then calculated by measuring the fraction of simulation runs in which the entire community persisted (Σ_*k*_*X*_*ki*_ > 10^−13^ for all *i*) after a sufficiently long time (t = 2 × 10^3^, which corresponded to the time taken for community persistence to reach an asymptote) in 500 runs. In this model, local stability analysis does not capture the model behavior, because even if the coexistence equilibrium (all variables have positive equilibrium) is locally stable, some species can lose the infected population depending on the initial conditions.

## Supplementary Information

Supplementary Information.

## Data Availability

All data generated and analyzed during this study are included in this published article.
