# Supplementary material for: Infected food web and ecological stability
Source: Sci Rep. 2022 May 17;12:8139. doi: 10.1038/s41598-022-11968-1 (PMC9114373; doi:10.1038/s41598-022-11968-1)

Supplementary Information

Infected food web and ecological stability

Akihiko Mougi

Supplemental Text

A model with interspecific infection

I consider not only intraspecific infectious disease but also interspecific one. Interspecific interaction occurs through contacts with their infected prey resources. Then, the term $-\Sigma_n\beta_n X_{In} X_{Si}$ and $+\Sigma_n\beta_n X_{In} X_{Si}$ (where n represents prey resource) are added into Eqs. (1) and (2), respectively, in the interspecific infection model. The other points are same with the main model.

A model with free-living parasite

Consider another type of food web model with a free-living parasite. The parasite infects to a specialized host through a contact process, and produced by the host species. In addition, the parasite decreases by death and infection to the host species (incorporated into both susceptible and infected host individuals). The food web model with free-living parasites is defined by the following ordinary differential equations:

$$dX_{Si}/dt = (r_{Si}X_{Si} + r_{Ii}X_{Ii})(1 - X_i) + M_1X_{Si} + M_2X_{Ii} - d_iX_{Si} - \beta_iW_iX_{Si} + \gamma_iX_{Ii}, \quad (1a)$$

$$dX_{Ii}/dt = \beta_iW_iX_{Si} - (d_i + v_i)X_{Si} - \gamma_iX_{Ii} + M_3X_{Ii}, \quad (1b)$$

$$dW_i/dt = \lambda_iX_{Ii} - \mu_iW_i - \beta_iW_iX_i, \quad (1c)$$

where W_i is the abundance of parasite species i, λ_i is the increase rate of the parasite species i and μ_i is the death rate of the parasite species i. The other symbols are same with those of the main model (eqs. 1) in the text.

Here, I assume that the parasite dynamics are faster than host dynamics. Then, W_i simplifies to its equilibrium:

$$W^{*}_{i} = \lambda_i X_{\mathrm{I}i}/(\mu_i + \beta_i X_i). \qquad (2)$$

By introducing eq. (2) into (1a) and (1b), I have the simplified equations:

$$dX_{\mathrm{S}i}/dt = (r_{\mathrm{S}i}X_{\mathrm{S}i} + r_{\mathrm{I}i}X_{\mathrm{I}i})(1 - X_i) + M_1X_{\mathrm{S}i} + M_2X_{\mathrm{I}i} - d_iX_{\mathrm{S}i} - \beta_i\lambda_iX_{\mathrm{S}i}X_{\mathrm{I}i}/(\mu_i + \beta_iX_i) + \gamma_iX_{\mathrm{I}i}, \qquad (3a)$$

$$dX_{\mathrm{I}i}/dt = \beta_i\lambda_iX_{\mathrm{S}i}X_{\mathrm{I}i}/(\mu_i + \beta_iX_i) - (d_i + v_i)X_{\mathrm{S}i} - \gamma_iX_{\mathrm{I}i} + M_3X_{\mathrm{I}i}, \qquad (3b)$$

A model with vertical transmission

The food web model with a vertical transmission is defined by the following ordinary differential equations:

$$dX_{\mathrm{S}i}/dt = (r_{\mathrm{S}i}X_{\mathrm{S}i} + (1-\mathrm{p})r_{\mathrm{I}i}X_{\mathrm{I}i})(1 - X_i) + M_1X_{\mathrm{S}i} + M_2X_{\mathrm{I}i} - d_iX_{\mathrm{S}i} - \beta_iW_iX_{\mathrm{S}i} + \gamma_iX_{\mathrm{I}i}, \qquad (4a)$$

$$dX_{\mathrm{I}i}/dt = \mathrm{p}r_{\mathrm{I}i}X_{\mathrm{I}i}(1 - X_i) + \beta_iW_iX_{\mathrm{S}i} - (d_i + v_i)X_{\mathrm{S}i} - \gamma_iX_{\mathrm{I}i} + M_3X_{\mathrm{I}i}, \qquad (4b)$$

where p is the fraction of vertical transmission.

Supplemental Figures

Figure S1. Additional results in a case with higher virulence in Fig. 2a. Parameters are identical to those in Fig. 2a.

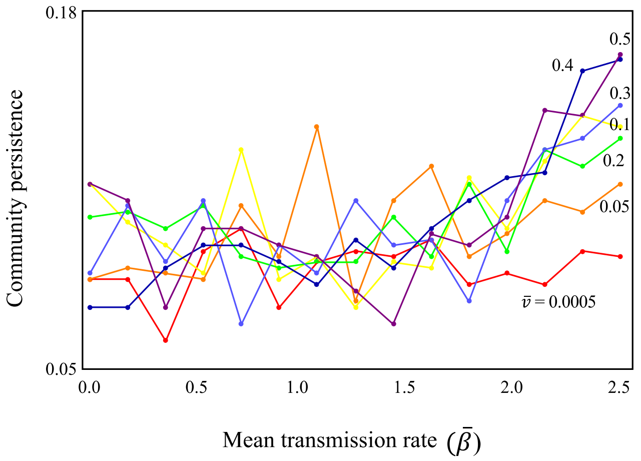

Figure S2. Effects of infertility on community stability. Each figure (a–f) has different combinations of mean virulence and mean infection rate, which are indicated in the right and upper side, respectively. (a–c) $\bar{v}$ = 0.05. (d–f) $\bar{v}$ = 0.005. Color represents the level of infertility. A_1 = 2. Other parameters are identical to those in Fig. 2.

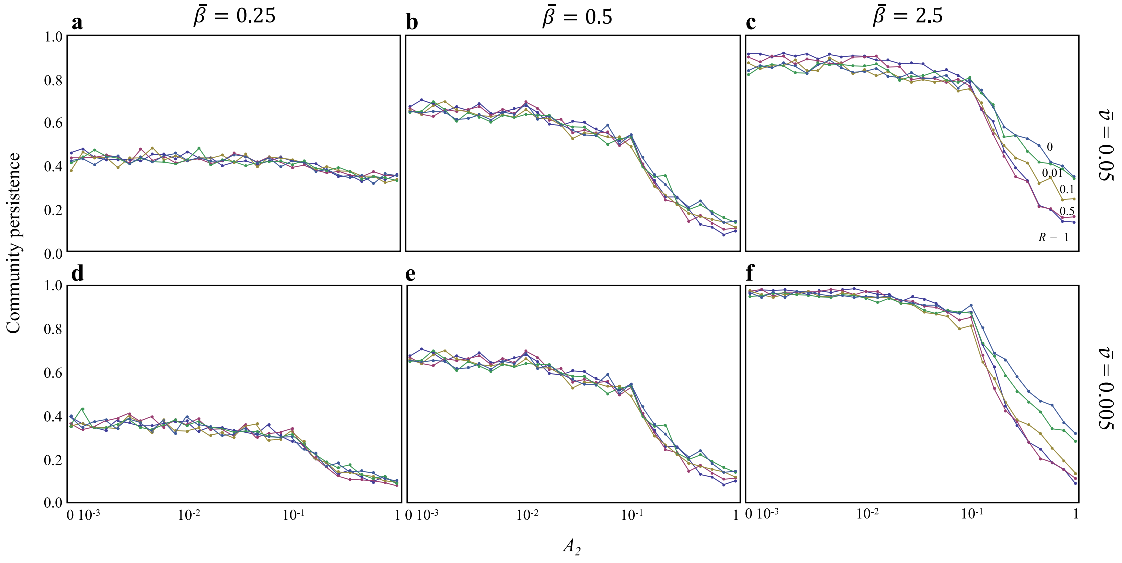

Figure S3. Effects of recovery rate on community stability. Color represents the mean recovery rate values. $A_1 = 2$. $\bar{v} = 0.05$. Other parameters are identical to those in Fig. 2.

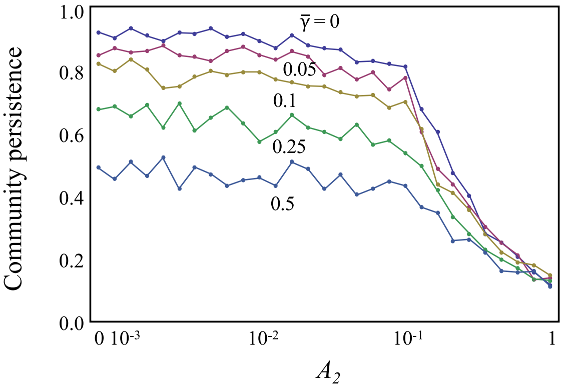

Figure S4. Complexity-stability relationship. (a, b) Effects of species richness. (a) C = 0.2. (b) C = 0.3. (c) Effects of connectance. N = 30. Colors represent food webs with or without parasites. Parameters are R = 0.1, A_1 = 1.5, A_2 = 0.01, $\bar{v}$ = 0.005, $\bar{\beta}$ = 2.5, $\bar{r}$ = 1, $\bar{d}$ = 0.005, $\bar{a}$ = 0.05, and $\bar{\gamma}$ = 0.005.

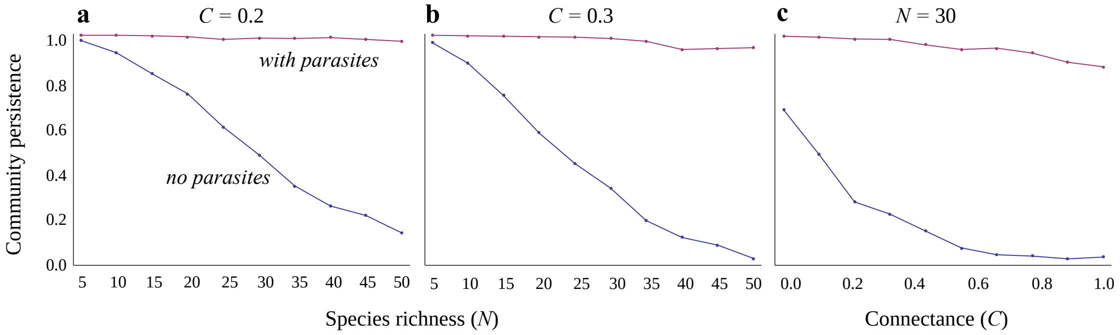

Figure S5. Effects of interspecific infection on community stability. Color represents mean interspecific infection rates, $\bar{\beta}'$ (see supplemental text for model details). Parameters are identical to those in Fig. 2g.

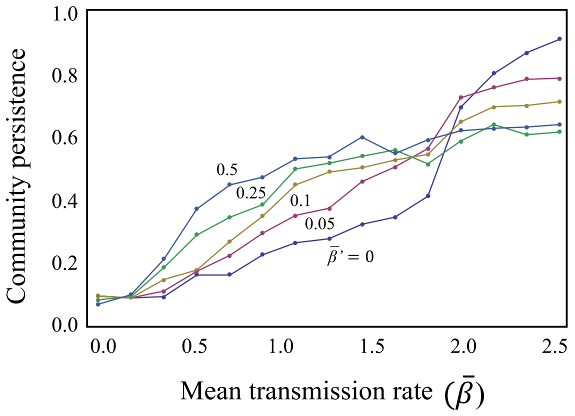

Figure S6. Effects of virulence and infection rate on community stability with varying interaction strengths in a random food web. Parameters are identical to those in Fig. 2.

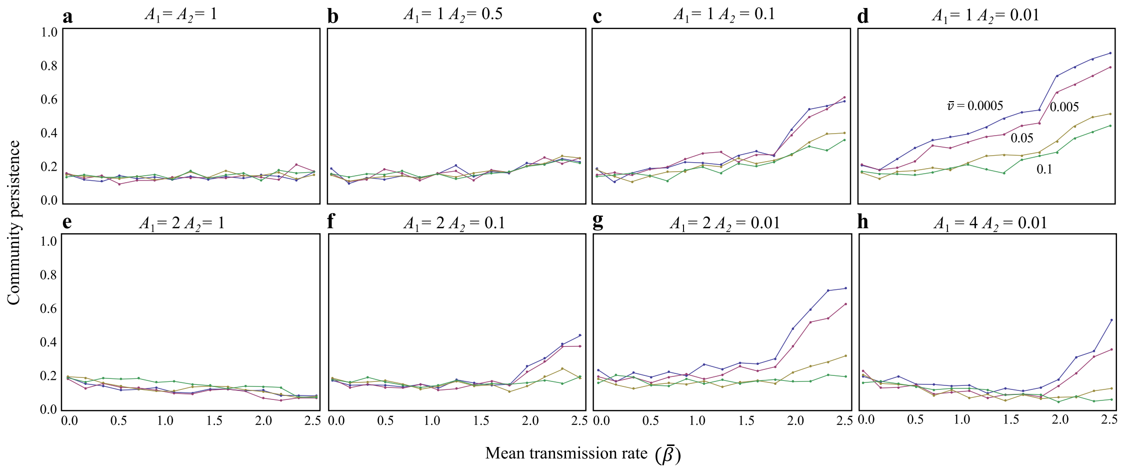

Figure S7. Effects of virulence and infection rate on community stability with varying interaction strengths in a free-living parasite model (see supplemental text for model details). $\bar{\lambda}$ = 5, $\bar{\mu}$ = 0.5. Other parameters are identical to those in Fig. 2.

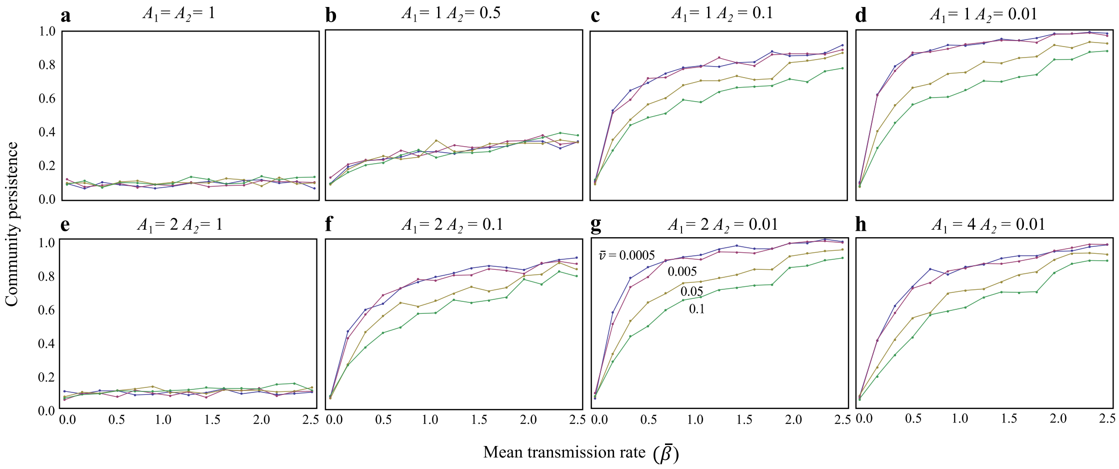

Figure S8. Effects of vertical transmission on community stability. See supplemental text for model details. N = 30 and C = 0.3. Parameters are identical to those in Fig. 2 except for A_1 = 2, $\bar{\beta}$ = 2.5 and $\bar{v}$ = 0.05 in (a) and A_1 = 2, A_2 = 0.01 and $\bar{v}$ = 0.05 in (b).

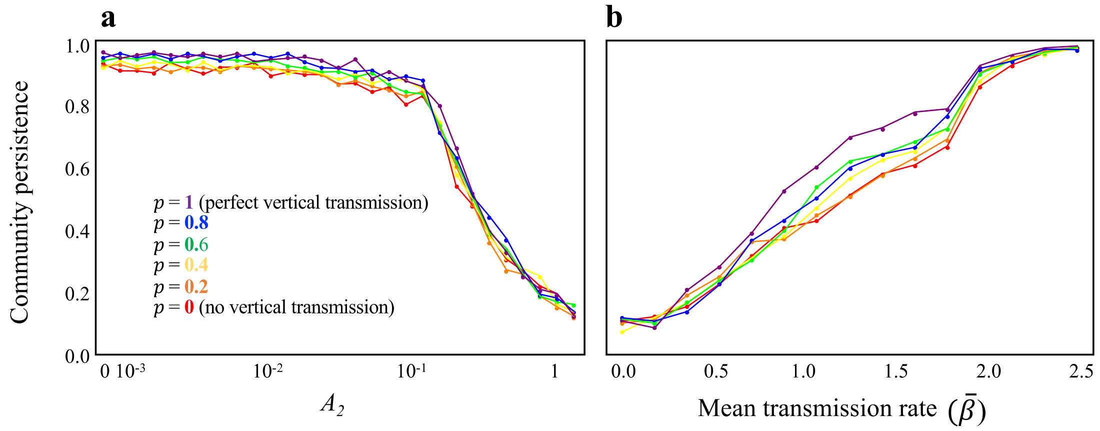

Supplement: Supplementary file 1 — Supplementary Information. [file 41598_2022_11968_MOESM1_ESM.pdf]
